# Crosstalk Within the Intestinal Epithelium: Aspects of Intestinal Absorption, Homeostasis, and Immunity

**DOI:** 10.3390/biomedicines12122771

**Published:** 2024-12-05

**Authors:** Liang-En Yu, Wen-Chin Yang, Yu-Chaun Liang

**Affiliations:** Agricultural Biotechnology Research Center, Academia Sinica, Taipei 115201, Taiwan; liangen@gate.sinica.edu.tw (L.-E.Y.); wcyang@gate.sinica.edu.tw (W.-C.Y.)

**Keywords:** enteroendocrine cells, Paneth cells, absorption, intestinal homeostasis, immunity, gut health

## Abstract

Gut health is crucial in many ways, such as in improving human health in general and enhancing production in agricultural animals. To maximize the effect of a healthy gastrointestinal tract (GIT), an understanding of the regulation of intestinal functions is needed. Proper intestinal functions depend on the activity, composition, and behavior of intestinal epithelial cells (IECs). There are various types of IECs, including enterocytes, Paneth cells, enteroendocrine cells (EECs), goblet cells, tuft cells, M cells, and intestinal epithelial stem cells (IESCs), each with unique 3D structures and IEC distributions. Although the communication between IECs and other cell types, such as immune cells and neurons, has been intensively reviewed, communication between different IECs has rarely been addressed. The present paper overviews the networks among IECs that influence intestinal functions. Intestinal absorption is regulated by incretins derived from EECs that induce nutrient transporter activity in enterocytes. EECs, Paneth cells, tuft cells, and enterocytes release signals to activate Notch signaling, which modulates IESC activity and intestinal homeostasis, including proliferation and differentiation. Intestinal immunity can be altered via EECs, goblet cells, tuft cells, and cytokines derived from IECs. Finally, tools for investigating IEC communication have been discussed, including the novel 3D intestinal cell model utilizing enteroids that can be considered a powerful tool for IEC communication research. Overall, the importance of IEC communication, especially EECs and Paneth cells, which cover most intestinal functional regulating pathways, are overviewed in this paper. Such a compilation will be helpful in developing strategies for maintaining gut health.

## 1. Introduction

Research into the gastrointestinal tract (GIT) has increased dramatically over recent decades. Globally, 88.99 million disability-adjusted life years were caused by digestive diseases in 2019, which constituted a significant burden worldwide [1]. The GIT communicates with other organs through the gut–brain, gut–muscle, gut–kidney, and gut–liver axes. Research shows that dysregulation of the GIT might result in diseases beyond the GIT. For example, we previously reviewed the relationship between neurological disorders, psychiatric disorders, and enteroendocrine cells (EECs), a type of intestinal epithelial cell (IEC) [2]. Furthermore, in agricultural animals, due to the limitations placed on the use of antimicrobial growth promoters, maintaining gut health without antibiotics has become a crucial topic in veterinary medicine [3].

Intestinal functions include digestion, absorption, barrier function, immune regulation, hormone secretion, and the repair and turnover of IECs, which involves proliferation and differentiation. Although the primary purpose of the GIT in animals is to acquire nutrients for the bioreaction requirements of the whole body, numerous studies suggest that the GIT also serves to defend the entire body from the invasion of pathogens via its tight junction structure, mucus layer, and antimicrobial peptides [4,5,6]. Furthermore, the hormones released from EECs modulate the functions and behavior of other tissues, such as regulating appetite in the brain, adipogenesis in adipose tissue, myogenesis in muscle, and hepatic steatosis in the liver [7,8,9]. Since intestinal functions are varied and widely impact the whole body, regulating intestinal functions is extremely complicated.

Gastrointestinal tract function alteration is highly dependent on the homeostasis of IECs, the composition of the microbiota, and signaling from other types of cells, such as immune cells. Most IECs can be divided into six types: enterocytes, goblet cells, tuft cells, EECs, Paneth cells, and intestinal epithelial stem cells (IESCs) [10]. The villus region contains mostly mature IECs, including enterocytes, goblet cells, tuft cells, and EECs, while the crypt area contains mostly immature IECs, with the majority being Paneth cells and IESCs in normal intestinal structures. Each type of IEC contributes a different intestinal function or collaborates to maintain the same intestinal function. Enterocytes are key absorbers that obtain nutrients in the lumen and transport nutrients to the whole body via the circulatory system. Goblet cells are responsible for secreting mucin and creating a protective mucus layer while also maintaining the intestinal tight junction in collaboration with other types of IECs, such as enterocytes or EECs [11]. EECs and tuft cells have a secretory cell lineage, sense material in the lumen, and activate the downstream responses, regulating intestinal barrier function, absorption, homeostasis, and immunity. IESCs are responsible for maintaining intestinal homeostasis through cell proliferation and differentiation, functions which are primarily supported by Paneth cells. The crosstalk between the microbiota and IECs has been found to affect the growth, maturation, and function of IECs, while IECs secrete antimicrobial peptides or other signals to shape the microbiota [12,13,14,15]. For example, the dysfunction of IEC mitochondria leads to gut microbiota dysbiosis, which might contribute to the pathogenesis of depression [16]. Cytokines derived from cells are another factor that influences intestinal functions. Interlukin-10 (IL-10) secreted from macrophages increases the proliferation of IECs and intestinal barrier function [4,17,18]. Although all three factors influence intestinal functions and have been well reviewed recently, the GIT functional alterations caused by the communication between different IECs, which might be among the first changes to affect intestinal functions, are less reviewed.

EECs and Paneth cells, two secretory cell lineages derived from intestinal epithelial stem cells (IESCs), are the most likely to be the activators of communication among IECs for regulating intestinal functions. EECs are chemosensory cells in the intestinal epithelium that monitor and sense the luminal content to secrete incretins, such as glucagon-like peptide 1 (GLP1) and glucagon-like peptide 2 (GLP2). GLP2 has been found to enhance nutrient transporter expression in enterocytes, a major absorptive cell lineage, while GLP1 analog treatment increases intestinal barrier function [19,20]. In mammals, Paneth cells have a close physical association with IESCs, as Paneth cells are located at the bottom of crypts adjacent to IESCs [21,22]. The solid physical association in structure between Paneth cells and IESCs increases the possibility of regulation between the two types of cells.

This article reviews the current understanding of and newly published evidence regarding the communication between different types of IECs that regulate intestinal functions, mainly focusing on EECs and Paneth cells. It should be noted that most of the study results were derived from animal models, mainly based on genetically modified rodents. Communication among IECs is classified based on aspects of intestinal functions. A clear overview of the cellular interaction between IECs will be a helpful reference for developing future strategies against digestive diseases and for maintaining gut health, including for applications such as enhancing agricultural animal production.

## 2. Intestinal Absorption: Enteroendocrine Cells and Enterocytes

Absorption of nutrients is a crucial feature of the GIT. Carbohydrates, amino acids, and fatty acids are the major nutrients that must be transported from the lumen into the apical side of IECs and further out of the basolateral side of IECs. Glucose is mainly absorbed via sodium-dependent glucose co-transporter 1 (SGLT1) and glucose transporter 2 (GLUT2). In contrast, amino acids are absorbed through multiple transporters, such as sodium-dependent neutral amino acid transporter B(0)AT1, which mediates the resorption of neutral amino acids, and excitatory amino acid transporter 3 (EAAT3), which mediates the uptake of excitatory amino acids [23]. The absorption of fatty acids is a relatively complex process and depends on the type of fatty acids. For example, long-chain fatty acids (LCFAs) and monoglycerides can combine with bile salts, which are derived from the gallbladder, to become micelles and cross the membrane of IECs, while middle-chain fatty acids (MCFAs), short-chain fatty acids (SCFAs), and glycerol can passively diffuse through the membrane of IECs. Moreover, fatty acids can pass through the membrane of IECs via protein-mediated transporters, such as fatty acid transport protein 4 (FATP4) [24,25,26]. Enterocytes are the absorptive cell lineage derived from IESCs that contain digestive enzymes on the surface of apical-side peptidase or sucrase. Enterocytes are highly abundant, making up around 70% of the total cells in the human ileum [27]. The nutrient transporter gene expression is highly dynamic and regulated, especially the difference between the villus and crypt areas. For example, the expression of B0AT1 in crypt-area enterocytes is inhibited by repressive transcriptional control elements, while these elements are removed in villus-area enterocytes to enhance B0AT1 expression for the absorption of amino acids [28]. Furthermore, the activity and expression of SGLT1 are upregulated in response to elevated intake of carbohydrates or luminal monosaccharides [29]. These findings suggest that the absorption function may be regulated by other factors.

**Figure 1:**
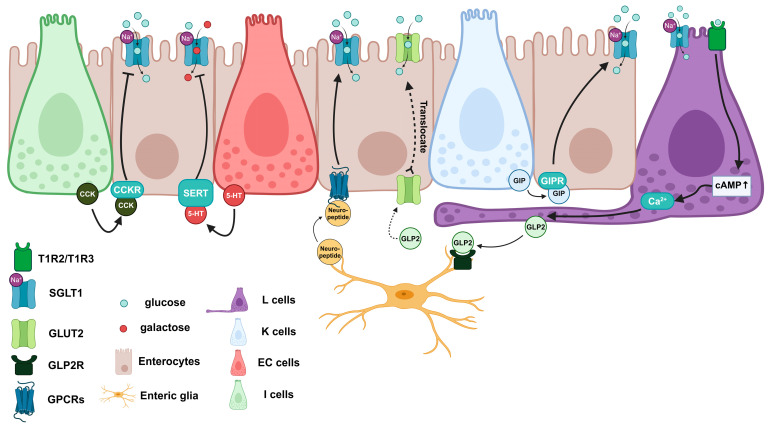
Intestinal glucose absorption is regulated via the communication between EEC-derived incretins and enterocytes. 5-HT, serotonin; cAMP, cyclic adenosine monophosphate; CCK, cholecystokinin; CCKR, cholecystokinin receptor; EC cells, enterochromaffin cells; GIP, gastric inhibitory peptide; GIPR, gastric inhibitory peptide receptor; GLP2, glucagon-like peptide 2; GLP2R, glucagon-like peptide 2 receptor; GLUT2, glucose transporter 2; GPCRs, G protein-coupled receptors; SERT, serotonin transporter; SGLT1, sodium glucose co-transporter 1; T1R1/T1R3, taste receptor type 1 member 2/taste receptor type 1 member 3. ↑, increase.

In glucose absorption regulation, EECs are able to modulate the expression of SGLT1 in enterocytes (Figure 1) [30]. EECs are the major sensors among IECs for detecting the nutrients in the lumen and secreting incretin to regulate multiple intestinal functions. L cells, a subtype of EECs, sense the glucose in the lumen via SGLT1 or T1R2/T1R3 sweet receptor, present on the apical side of L cells, followed by the secretion of GLP2 through increasing the cytosol calcium concentration by cAMP elevation or calcium release from the mitochondria [2,31]. L cells, GLP2-producing cells, contain axon-like basal processes called neuropods [32]. GLP2 is secreted from neuropods to activate the GLP2 receptor (GLP2R) on enteric glia cells, followed by secreting a neuropeptide that increases SGLT1 expression in enterocytes [31,33,34,35]. Similarly, K cells, a subtype of EECs, release glucose-dependent insulinotropic polypeptide (GIP) to enhance glucose absorption [36]. Furthermore, GLP2 promotes the translocation of GLUT2, which turns the GLUT2 from the basolateral membrane to the brush border membrane, temporarily dramatically enhancing glucose absorption [31,37]. On the other hand, enterochromaffin cells (ECs), a subtype of EECs, secrete serotonin, which inhibits D-galactose absorption [38]. In addition, I cells, a subtype of EECs, secrete cholecystokinin (CCK) to inhibit SGLT1 expression, downregulating glucose uptake from the brush border membrane [39,40]. However, knowledge of the detailed mechanisms of EEC regulation of glucose absorption is limited, and further investigation is required.

**Figure 2:**
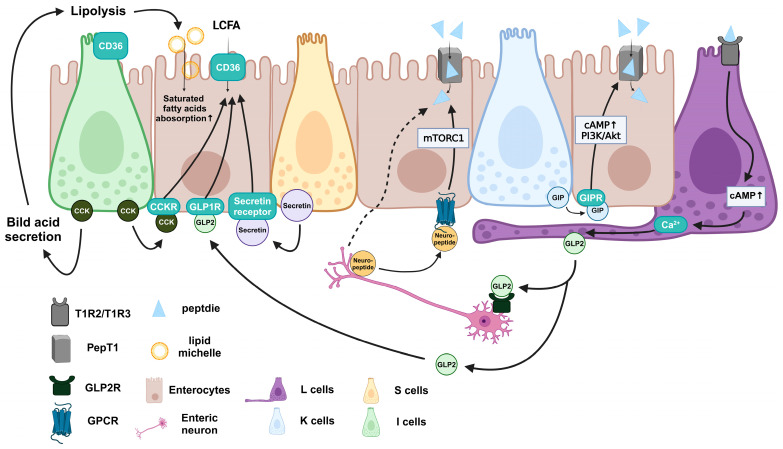
Intestinal amino acid, peptide, fatty acid, and lipid absorption are regulated via the communication between EEC-derived incretins and enterocytes. cAMP, cyclic adenosine monophosphate; CCK, cholecystokinin; CCKR, cholecystokinin receptor; CD36, cluster of differentiation 36; GIP, gastric inhibitory peptide; GIPR, gastric inhibitory peptide receptor; GLP2, glucagon-like peptide 2; GLP2R, glucagon-like peptide 2 receptor; GPCRs, G protein-coupled receptors; LCFA, long-chain fatty acid; mTORC1, mammalian target of rapamycin complex 1; PepT1, intestinal peptide transporter 1; PI3K/Akt, phosphatidylinositol 3-kinase/protein kinase B. ↑, increase.

In amino acid and peptide absorption, peptide transporter 1 (PepT1) is a major transporter that is modulated by incretins (Figure 2). GIP enhances PepT1 expression via cAMP and the phosphatidylinositol 3-kinase/protein kinase B (PI3K/ATK) pathway in enterocytes [41]. Furthermore, GLP2 treatment elevates lysine, threonine, valine, isoleucine, methionine, and phenylalanine absorption in mice [42]. A study on GLP2 receptor (GLP2R)-knockout mice confirmed that an increase in amino acid absorption requires the enteric nervous system and mammalian target of rapamycin complex 1 (mTORC1) signaling [42].

EECs regulate lipid and fatty acid absorption in multiple ways (Figure 2). CCK is associated with lipid absorption by regulating bile acid secretion [43,44]. Furthermore, CCK-knockout mice are resistant to high-fat-diet-induced obesity by exhibiting decreased triglyceride absorption and body weight gain [43]. Regarding protein-mediated transporters, CD36 mediates LCFA absorption and is associated with fatty acid uptake via dynamic palmitoylation-regulated endocytosis [45,46]. Although CD36 knockout does not influence triglyceride absorption in mice, CD36-knockout mice exhibit decreased fatty acid and cholesterol absorption [47,48]. CD36 has been found to express on EECs to sense fatty acids for CCK secretion [49,50]. Furthermore, CCK and secretin, another type of incretin derived from EECs, can enhance CD36 expression on enterocytes, increasing fatty acid absorption [51,52]. GLP2 also increases CD36 on IECs, enhancing fatty acid absorption, which requires the presence of GLP1 receptor (GLP1R) [50,53]. In contrast, although it has not been found in IECs, GLP1 inhibits CD36 expression in macrophages and cardiomyocytes, which indicates the potential of GLP1 to decrease CD36 in IECs [54,55].

In contrast with the normal physical regulation of incretins derived from EECs, in dysregulated absorption and disease, treatment with peptide YY (PYY), an incretin secreted from L cells, restores electrophysiological reactions to enhance glucose and peptide absorption in EEC-deficient mice [56]. A similar study found that EEC-deficient mice showed impairment of lipid absorption and decreased growth performance with diarrhea [57]. In this specific situation, restoring nutrient absorption can potentially improve the postnatal survival rate and diarrhea symptoms in patients with severe congenital malabsorptive diarrhea [56,57]. Although the phenomenon of absorption regulation among IECs has been found, further investigation is needed to reveal the detailed mechanisms and features, especially with regard to the absorption of amino acids and fat.

## 3. Intestinal Niche: Paneth Cells, Enteroendocrine Cells, Tuft Cells, Enterocytes, and Intestinal Epithelial Stem Cells

IESCs are a fundamental component of the intestinal epithelium. Optimal intestinal epithelial functions rely on the composition of the epithelium population, which is primarily determined by the proliferation and differentiation of IESCs [58,59]. Leucine-rich repeat-containing G-protein coupled receptor 5 (LGR5) plays a crucial role in maintaining the functional integrity and structure of IESCs via R-spondin-triggered Wnt/β-catenin signaling [60,61,62]. IESC proliferation and differentiation are highly regulated functions mainly controlled by bone morphogenetic protein (BMP) and the Wnt pathways [63]. At the intestinal niche, the enrichment of BMP ligands and suppression of Wnt signaling create a high-BMP-signaling environment that promotes the proliferation, differentiation, and replication of IESCs [64,65].

The intestinal niche is a microenvironment composed of IESCs and Paneth cells at the bottom of the crypt, which provides signals for IESC maintenance, regulation, and activation via releasing growth factors, such as epidermal growth factor (EGF), transforming growth factor (TGF), Wnt3, and delta-like canonical Notch ligand 4 (Dll4), which activates Notch signaling [21,61,66,67,68]. Furthermore, Paneth cells are responsible for metal uptake and preserve barrier function [22]. The intestinal niche is extremely sensitive to a change in diet, such as a high-fat or high-carbohydrate diet, or calorie restriction/fasting [69,70,71]. Furthermore, the rate of glycolytic flux can impact IESC function [72]. However, due to the structure of the villus and crypts, IESCs have been thought to not sense the lumen’s components immediately. Instead, EECs are likely to sense the material in the lumen, since EECs are major sensing cells in IECs. Indeed, the absence of EECs induces energy expenditure and increases IESC activity and the fasting reaction of IESCs in mice [73]. Furthermore, dedifferentiation (the reversion of differentiated cells within its own lineage) into IESCs has been found in Paneth cells and EECs [74,75,76,77]. Therefore, EECs and Paneth cells are most likely to communicate and influence IESC activity and behavior.

**Figure 3:**
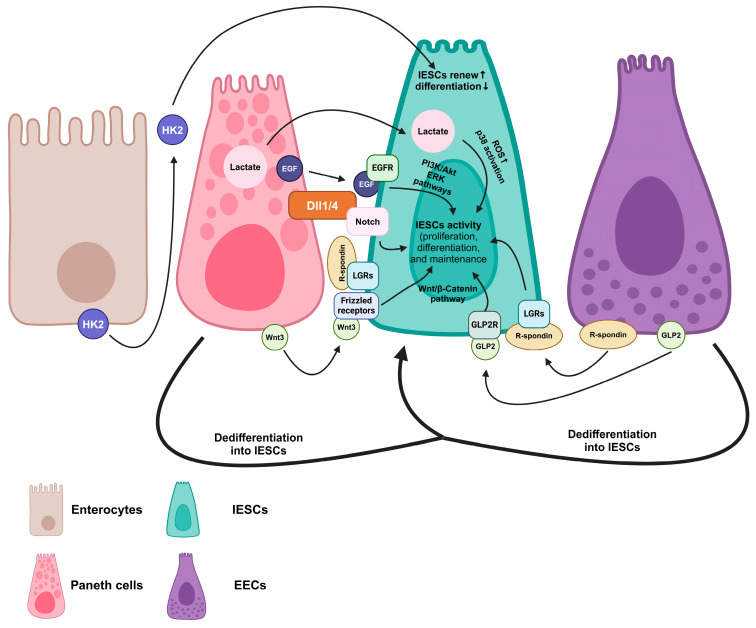
IESC activity, including proliferation, differentiation, and maintenance, is regulated via the communication between EECs, Paneth cells, enterocytes, and IESCs. Dll1/4, delta-like canonical Notch ligand 1/4; EECs, enteroendocrine cells; EGF, epidermal growth factor; EGFR, epidermal growth factor receptor; GLP2, glucagon-like peptide 2; GLP2R, glucagon-like peptide 2 receptor; HK2, hexokinase 2; IESCs, intestinal epithelial stem cells; LGRs, leucine-rich repeat containing G protein-coupled receptors; p38, p38 mitogen-activated protein kinase; PI3K/Akt, phosphatidylinositol 3-kinase/protein kinase B; ROS, reactive oxygen species. ↑, increase; ↓, decrease.

Wnt3, a crucial protein regulating intestinal homeostasis, produced by Paneth cells, can be amplified via R-spondin 1 and has spatial specificity, which means that Wnt3 cannot freely diffuse but directly contacts the Frizzled receptors to promote IESC activity (Figure 3) [78]. EGF is another factor that modulates the activity of IESCs via EGF receptor (EGFR) expression in IESCs. Similar to Wnt3, the secretion of EGF in Paneth cells can activate EGFR signaling to regulate IESC proliferation with location specificity [79]. Notch signaling ligands, such as Dll4, are expressed on the membrane of Paneth cells and drive the initiation of absorptive cells’ progenitor differentiation via Hes1 expression [80,81,82]. Lactate, a fermentative product derived from the reduction of pyruvate, the end product of the glycolytic pathway, can be transferred from Paneth cells into IESCs and converted into pyruvate to increase reactive oxygen species (ROS) signaling, p38 activation, and IESC differentiation [79,83]. Furthermore, communication between Paneth cells and IESCs can be triggered by microbiota, such as *Bifidobacterium* spp. and *Lactobacillus* spp., via increasing Wnt3a products in Paneth cells to activate Wnt/β-catenin signaling in IESCs [84]. Interleukin 22 (IL22) treatment in coculture of Paneth cells and IESCs enhances the activity of IESCs via interleukin 22 receptor subunit α1 (IL22RA1)/signal transducer and activator of transcription 3 (STAT3) signaling [85,86,87].

EECs can sense the nutrients present in the lumen to modulate crypt metabolism (Figure 3). In modified EEC-deficient mice, the activity of IESCs increases with fatty acid oxidation and mitochondrial activity while decreasing mammalian target of rapamycin (mTOR) signaling, which are important for the proliferation and differentiation of IESCs [73,88,89,90]. GLP2 has been found to enhance the proliferation of IESCs at the length of villi by stimulating the S-phase entry of IESCs [91]. Furthermore, GLP2 treatment promotes microvillus length and the proliferation of IESCs via intestinal epithelial insulin-like growth factor 1 receptor (IE-IGF1R) [91,92]. In silicon-rich alkaline mineral water-fed piglets, the release of GLP2 is increased, which activates Wnt/β-catenin signaling and regeneration in IESCs [93]. Upon Paneth cell depletion in mice, EECs have been found to alternatively support IESCs via Notch signaling activation with location shifting, altering the EEC-enriched region from the villus to the bottom of the crypt area [94]. The potential mechanism of Notch signaling activation and location shifting might be through R-spondin derived from EECs and differentiation behavior alteration in IESCs [60,95]. In response to the change in the luminal environment, germ-free conditions increased the differentiation of 50% of IESCs toward EECs in flies [15]. Furthermore, normal microbiota elevated IESC differentiation into enterocytes via the Imd/Relish pathway, and pathogenic microbiota led to the differentiation of IESCs into EECs via activation of Janus kinase/signal transducers and activators of transcription (JAK-STAT) signaling in flies [15]. The evidence suggests communication between sensing cells, the majority of EECs, and IESCs, which implies that the luminal materials influence the functions of IESCs via the intestinal paracrine pathway.

Beyond Paneth cells and EECs, the absorptive lineage of IECs, enterocytes, also affect the regulation of the activity of IESCs (Figure 3). Hexokinase 2 (HK2) is a target gene of Wnt signaling in IECs, and mature enterocytes are major expressors of HK2, at around 3- to 4-fold higher levels than EECs, goblet cells, and immature enterocytes [96,97]. The activity of IESCs differentiated into Paneth cells, goblet cells, and EECs is increased via the activation of p38 mitogen-activated protein kinase and the increase in transcriptional factor atonal homolog 1 expression in HK2-knockout mice [72]. Furthermore, the renewal ability of IESCs was decreased in HK2-knockout mice, which suggests that there is communication between enterocytes and IESCs [72]. Transcription factor c-Maf is expressed in differentiated lower- and mid-villus enterocytes. The inactivation of c-Maf elevates glucose absorption while suppressing amino acid and lipid absorption [98]. Furthermore, loss of c-Maf induces gut remodeling and tuft cell expansion [98,99]. Therefore, the proliferation and differentiation of IESCs can be regulated by environmental changes in the lumen via Paneth cells, EECs, and enterocytes. However, understanding of the communication between IECs in modulating IESC functions has not been thoroughly investigated. Future research is needed to reveal more factors and mechanisms for developing strategies for promoting IESC activity.

## 4. Intestinal Immune Regulation: Cytokines, Enteroendocrine Cells, Goblet Cells, and Tuft Cells

Regulation of intestinal immunity is usually discussed in conjunction with disease and involves multiple components, particularly immune cells and microbiota metabolites. In colonic-inflammation-induced ulcerative colitis, for example, the deficiency in regulator of G-protein signaling 10 (RGS10) suppressed intestinal inflammation via inhibition of T helper cells 1 (Th1) and T helper cell 17 (Th17)-mediated immune responses [100]. Furthermore, the vagus nerve plays a role in inflammatory bowel disease (IBD) by elevating the anti-inflammatory effects via acetylcholine and its receptor [101,102,103]. Recent review papers have discussed the modulation of fungi and probiotics chassis (reusable biological frames containing ideal probiotics) in IBD [104,105,106]. Moreover, products secreted from EECs have been found to affect the regulation of immunity in the gut. GLP2 treatment alleviates the lipopolysaccharide (LPS)-induced inflammatory response in pigs and rats [107,108,109]. Furthermore, the activation of GLP2 receptor (GLP2R) decreases the LPS-induced inflammation and expression of cytokines derived from macrophages [108]. However, the present paper focuses on the communication between IECs, which involve immune functions. Therefore, the regulation axis beyond IECs will not be discussed.

Cytokines are related to the regulation of intestinal barrier integrity, inflammation, and repair when facing damage or severe inflammatory response. IECs can secrete cytokines to modulate the inflammation response, although most cytokines come from immune cells, such as macrophages and innate lymphoid cells. Interleukin-6 (IL6), -7 (IL7), -10 (IL10), -15 (IL15), -17 (IL17), -18 (IL18), -23 (IL23), -25 (IL25), -33 (IL33), interleukin 1 beta (IL1β), and transforming growth factor beta (TGFβ) can be produced by IECs (Table 1) [4,110,111]. Among these cytokines, IL6, IL10, IL17, IL18, IL23, IL33, and TGFβ can target IECs to modulate intestinal function and immunity, while IL7, IL15, and IL25 target other cell types but not IECs [112,113,114,115,116,117]. Some cytokines, such as IL10, have been proposed to act via the autocrine or paracrine pathway, suggesting that cytokines might be used as communicators between IECs [118]. IL6 increases GLP1 secretion from EECs and stimulates the proliferation and repair of IESCs after acute injury, while it is dispensable in normal physical conditions [119,120,121]. In vitro experiments indicate that activating the IL6/STAT3 pathway damages the intestinal barrier function with P2Y purinoceptor 13 (P2RY13) treatment [122,123]. Moreover, a clinical study shows that the IL6/STAT3 signaling pathway disrupts intestinal barrier integrity in ulcerative colitis patients via histone 3 lysine 27 acetylation (H3K27ac) [124]. Macrophage-derived IL10 enhanced the repair of IECs via WISP-1 signaling during inflammation [17]. Since IL10 is also expressed in IECs, it is possible that IECs secrete IL10 to enhance the repair effect under inflammation. Indeed, both in vitro and in vivo studies show that deletion of IEC-specific IL10 receptors enhances the intestinal barrier damage and sensitivity of inflammatory-related diseases [125,126,127]. Furthermore, IL10 receptor depletion increases goblet cell differentiation and decreases absorptive cell lineage differentiation, while diminished IL10 signaling elevates proliferation under homeostatic conditions [128,129]. Mucus functions as a first-line innate defense, which is a component of first-line intestinal immunity. The major component of mucus is gel-forming mucin 2 (Muc2), which is derived from goblet cells. In the small intestine, a single layer of mucus is present, while an outer and inner mucus layer are formed in the colon [130]. In vitro studies show that IL17 treatment induces pyroptosis in IESCs and enterocytes, which can be limited by IL17-neutralizing antibody treatment [115,116]. Meanwhile, IL17 treatment induces differentiation into the secretory cell lineage in IESCs and mucin production in goblet cells [116,131]. Since IL17 can also be produced by immune cells, the bidirectional effects of IL17 might be dependent on the location of IL17. The deletion of IL18 or IL18 receptor (IL18R) in IECs induces protection from colitis and maintains barrier integrity in mice, which indicates that IL18 is crucial in driving the pathogenesis of inflammation-induced colitis [112]. The inhibition of IEC-derived IL18 is important to maintain the mucus layer and intestinal barrier function in colitis [4,112]. Overexpression of IL18 induces the loss of mature goblet cells and sensitivity to inflammation [112]. In contrast, IL18 promotes IESC activity via protein kinase B-transcription factor 4 (AKT-TCF4) signaling and the antimicrobial response of Paneth cells via STAT3, which suggests that the bidirectional effects of IL18 might be due to the location difference between villus and crypts [132]. Similarly, IL23 induces antimicrobial peptide expression in IECs [133]. The deletion of the IL23 receptor (IL23R) in IECs disrupts microbiota composition with an expansion of flagellated bacteria and elevated death rate caused by colitis, which suggests that IL23 has a protective effect in inflammation-induced colitis [134,135]. IL33 can promote mucin production in IECs by increasing goblet cell differentiation via activating the receptor suppression of tumorigenicity 2 (ST2) [136,137,138,139]. IL33 activates cytokine expression to influence intestinal immunity in tuft cells [140]. In *H. polygyrus* infection, the parasite releases an inhibitor to silence the immune response from tuft cells via limiting IL33 secretion from injured IECs [141]. Antimicrobial peptide expression can be elevated by IL33 in Paneth cells [142]. Furthermore, IL33 targets IESCs to impact regeneration ability and stemness under inflammation via the IL33-EGF pathway [137,143,144]. TGFβ enhances intestinal barrier integrity by regulating plasticity, proliferation, and differentiation in IESCs [145,146]. The terminal differentiation of villus tip enterocytes is decided by distal TGFβ signaling, and TGFβ activates IESC intestinal differentiation, which is negatively regulated by microRNA-146b via the ERK1/2 pathway [99,113]. Furthermore, in mothers against decapentaplegic homolog 4 (Smad4)-deficient mice, TGFβ treatment elevates intestinal inflammation, which indicates that TGFβ suppresses intestinal inflammation via the TGFβ/Smad4 axis [147]. Although most studies of intestinal cytokines focus on the relationship between immune cells and IECs, the communication between IECs through cytokines as mediators is important for regulating intestinal immunity and homeostasis, especially crucial with specific location due to the unique structure in the intestine.

With regard to other factors, GLP2 regulates the immune response of Paneth cells to reduce intestinal inflammation by increasing the expression of IL10 and lysozyme and decreasing IL6 [148]. GLP1R-knockout mice exhibit a reduced number of goblet cells, lose colonic epithelial integrity, and die before 20 weeks of age in a germ-free environment, which indicates the importance of communication among EECs, IESCs, and goblet cells via GLP1R signaling [149]. Prostaglandin D2 (PGD2), derived from tuft cells, promotes mucin secretion in goblet cells [150]. Recent studies show that local glucose stimulates the change in the behavior of goblet cells with regard to mucin secretion and maintaining barrier integrity [151,152]. Considering the expression level of glucose transporters in IECs, glucose may stimulate other types of IECs to influence goblet cell behavior, although the mechanism is unknown.

## 5. Tools for Investigating IEC Communication

Investigating communication between IECs is relatively hard due to the complexity of different cell types, dynamic functions, and the unique structures of the IECs. As a result, currently, there is a limited number of appropriate models and tools. In vivo experiments can quantify nutrient absorption, barrier function, and IESC activity via BrdU intraperitoneal injection [153,154]. Animal serum is used for the indirect measurement of secreted incretins via the endocrine pathway [155]. Gene-knockout mice are widely used for investigating the functions of specific genes. Indeed, most research focused on communication between IECs used in vivo animal models and in vitro enteroid models [79]. However, in vivo experiments cannot directly detect the secretion function of incretins, since the incretins might be used in multiple tissues or generated from other tissues.

As mentioned above, EECs have been thought to be the major communicators among IECs. However, the function, communication, and working mechanism of EECs are challenging to quantify efficiently. Unlike regular nutrient transporters, which require gene expression for their function, the incretins of EECs are prepared and stored in the EEC cytosol before secretion to have an immediate response. Therefore, although the incretin mRNA and protein expression might be changed by treatment, it might not reflect an actual functional alteration of incretins, which requires incretins to be released and bind to the targeting receptors. Ex vivo experiments, such as the Ussing chamber, have been used to measure intestinal absorption, quantify intestinal permeability, and potentially detect the direct secretion function of incretins, which are secreted via the endocrine pathway [156,157,158].

Although in vitro 2D models can generate the luminal and serosal side of cells with the transwell system, 2D cell models lack a 3D structure, which means they fail to capture the importance of spatiality. Furthermore, the effect of cell–cell communication is absent in 2D cell models based on a single cell line. To solve these problems, an intestinal organoid model has been developed, called enteroids. Enteroids are self-organized 3D structures that recapitulate the identity of the original tissue to a certain degree. There are two types of enteroids, basolateral-out enteroids derived from IESCs or intestinal niches and apical-out enteroids derived from embryonic IESCs or basolateral-out enteroids by controlling the polarity of enteroids [10,159,160,161]. Since enteroids contain all types of IECs and, relatively-speaking, maintain intestinal 3D structure, this model is suitable for investigating the communication between IECs. Furthermore, the enteroid model has been proposed as an ideal model for investigating rare IECs, such as tuft cells and EECs [162,163,164].

From the perspective of immunity, the direct quantification of mucus is challenging, as mucus harvested from the laboratory and clinical surgery might be contaminated by materials transiting through the intestinal lumen, apoptosis epithelium, or other cell components [165,166]. Recently, an ex vivo method was developed to quantify intestinal mucus [167]. HT29-MTX, goblet cells line, Caco-2 cells, and intestinal epithelial cells from the large intestine were used to generate an in vitro mucus model [168]. However, the communication between IECs was lost in the model, since the cell types in the model were limited. The enteroid model has also been proposed to reveal the IEC cytokine-producing system [18]. Although cytokines can be produced in IECs, the specific cell types of IECs responsible for specific cytokines remain unclear. Therefore, the enteroid model might be beneficial not only for discovering the connection between cytokines and their specific products or targeting IECs but also for investigating the communication between IECs via cytokines.

## 6. Conclusions

The present review overviewed the communication between IECs, including intestinal absorption, intestinal niche homeostasis, and intestinal immunity. The network between EECs and enterocytes regulates intestinal absorption, while intestinal immunity and niche homeostasis are modulated by communication involving cytokines expressing IECs, EECs, Paneth cells, and IESCs. The current tools available for investigating IEC networks were discussed. Overall, the importance of IEC communication, especially modulation by EECs and Paneth cells, and the current understating of the IECs network was presented. This overview may be helpful in developing strategies for maintaining gut health.

## Figures and Tables

**Table 1:** Summary of IEC-derived cytokines with regard to targeting cells, functions, and pathways in IECs.

Cytokines	Producing Cells	Targeting Cells	Functions and Pathways in IECs
IL6	Unclarified IECs	Unclarified IECs EECs	GLP1 secretion ↑ Proliferation and repairment ↑ (after injury) IL6/STAT3 pathway → intestinal barrier integrity ↓
IL10	Unclarified IECs	Unclarified IECs	WISP-1 signaling → IECs repairment ↑ Intestinal barrier integrity ↑ Proliferation ↑ Skewing differentiation toward absorptive cell lineage
IL17	Unclarified IECs	IESCs Enterocytes Goblet cells	IESC pyroptosis ↑ Enterocyte pyroptosis ↑ Mucin ↑ Skewing differentiation toward secretory cell lineage
IL18	Unclarified IECs	Unclarified IECs Paneth cell IESCs	Intestinal barrier integrity ↓ Mucus layer ↓ Goblet cell number and maturation ↓ STAT3 pathway → antimicrobial peptide ↑ (in Paneth cells) PI3K/Akt/TCF4 pathway → proliferation ↑ (in IESCs)
IL23	Unclarified IECs	Unclarified IECs	Antimicrobial peptide ↑ Maintain microbiome composition
IL33	Unclarified IECs	Unclarified IECs IESCs Paneth cells Tuft cells	Mucin ↑ Goblet cell differentiation ↑ IL33/EGF pathway → regeneration and proliferation ↑ Antimicrobial peptide ↑ Cytokine expression in tuft cells ↑
TGFβ	Unclarified IECs	Unclarified IECs IESCs	Intestinal barrier integrity ↑ Proliferation ↑ Differentiation ↑

EECs, enteroendocrine cells; EGF, epidermal growth factor; GLP1, glucagon-like peptide 1; IECs, intestinal epithelial cells; IESCs, intestinal epithelial stem cells; IL6, interleukin 6; IL10, interleukin 10; IL17, interleukin 17; IL18, interleukin 18; IL23, interleukin 23; IL33, interleukin 33; PI3K/Akt, phosphatidylinositol 3-kinase/protein kinase B; STAT3, signal transducer and activator of transcription 3; TCF4, transcription factor 4; TGFβ, transforming growth factor beta; WISP-1, Wnt-inducible-signaling pathway protein 1; ↑, increase; ↓, decrease.

## Data Availability

Not applicable.
